# Intra-Scanner and Inter-Scanner Reproducibility of Automatic White Matter Hyperintensities Quantification

**DOI:** 10.3389/fnins.2019.00679

**Published:** 2019-07-10

**Authors:** Chunjie Guo, Kai Niu, Yishan Luo, Lin Shi, Zhuo Wang, Meng Zhao, Defeng Wang, Wan’an Zhu, Huimao Zhang, Li Sun

**Affiliations:** ^1^Department of Radiology, The First Hospital of Jilin University, Changchun, China; ^2^Department of Otorhinolaryngology Head and Neck Surgery, The First Hospital of Jilin University, Changchun, China; ^3^BrainNow Medical Technology Limited, Sha Tin, Hong Kong; ^4^Department of Imaging and Interventional Radiology, The Chinese University of Hong Kong, Sha Tin, Hong Kong; ^5^Department of Neurology and Neuroscience Center, The First Hospital of Jilin University, Changchun, China

**Keywords:** reproducibility of results, white matter, magnetic resonance imaging, brain, imaging, three-dimensional

## Abstract

**Objectives:** To evaluate white matter hyperintensities (WMH) quantification reproducibility from multiple aspects of view and examine the effects of scan–rescan procedure, types of scanner, imaging protocols, scanner software upgrade, and automatic segmentation tools on WMH quantification results using magnetic resonance imaging (MRI).

**Methods:** Six post-stroke subjects (4 males; mean age = 62.8, range = 58–72 years) were scanned and rescanned with both 3D T1-weighted, 2D and 3D T2-weighted fluid-attenuated inversion recovery (T2-FLAIR) MRI across four different MRI scanners within 12 h. Two automated WMH segmentation and quantification tools were used to measure WMH volume based on each MR scan. Robustness was assessed using the coefficient of variation (CV), Dice similarity coefficient (DSC), and intra-class correlation (ICC).

**Results:** Experimental results show that the best reproducibility was achieved by using 3D T2-FLAIR MRI under intra-scanner setting with CV ranging from 2.69 to 2.97%, while the largest variability resulted from comparing WMH volumes measured based on 2D T2-FLAIR MRI with those of 3D T2-FLAIR MRI, with CV values in the range of 15.62%–29.33%. The WMH quantification variability based on 2D MRIs is larger than 3D MRIs due to their large slice thickness. The DSC of WMH segmentation labels between intra-scanner MRIs ranges from 0.63 to 0.77, while that for inter-scanner MRIs is in the range of 0.63–0.65. In addition to image acquisition, the choice of automatic WMH segmentation tool also has a large impact on WMH quantification.

**Conclusion:** WMH reproducibility is one of the primary issues to be considered in multicenter and longitudinal studies. The study provides solid guidance in assisting multicenter and longitudinal study design to achieve meaningful results with enough power.

## Key Points

- The intra-scanner and inter-scanner WMH reproducibility study in the same cohort.
- The best reproducibility was achieved by using 3D T2-FLAIR MRI under intra-scanner setting.
- There is a large variability in comparing WMH quantification results based on 2D T2-FLAIR MRI with those of 3D T2-FLAIR MRI.

## Introduction

White matter hyperintensities, commonly found on T2-weighted T2-FLAIR brain MR images in the elderly, are associated with a number of neuropsychiatric disorders, including multiple sclerosis (MS) (Filippi et al., 2016), vascular dementia, Alzheimer’s disease (AD) (Fazekas et al., 1996; Hirono et al., 2000), mild cognitive impairment (DeCarli et al., 2001), stroke (Fazekas et al., 1993), and Parkinson’s disease (Marshall et al., 2006), and even in patients with primary mental disorders including mood disorders and schizophrenia spectrum disorders (Brown et al., 1995). Many studies have provided evidence that WMH have a strong impact on cognitive functioning (Gunning-Dixon and Raz, 2000) and they have been associated with impairment in a number of domains (Cees De Groot et al., 2000; Prins et al., 2004). WMH usually have a higher signal intensity compared to the normal-appearing white matter on FLAIR sequences and may appear iso- or hypointense on T1-weighted MR images. It can be measured quantitatively and non-invasively on large population samples and have been proposed as an intermediate marker, which could be used for the identification of new risk factors and potentially as a surrogate end point in clinical trials (Schmidt et al., 2004).

One challenging issue in studying WMH is the accurate and robust quantification and localization, given their variability and scattered spatial distribution. There are a number of automatic or semiautomatic methods and tools studying WMH segmentation and quantification, including thresholding method (Payne et al., 2002; Gibson et al., 2010; Simões et al., 2013), clustering methods (Admiraal-Behloul et al., 2005; Schmidt et al., 2012; Jain et al., 2015), and machine learning algorithms (Sweeney et al., 2014; López-Zorrilla et al., 2017; Rachmadi et al., 2018). While there are so many methods studying the accuracy of WMH segmentation and quantification, few studies examined the reproducibility of WMH quantification.

Accurate WMH quantification is of vital importance not only because it is associated with an increased risk of stroke, cognitive decline, dementia, and death, but also because their progression has been studied in association with cognitive decline, with increasing progression predicting a more rapid decline in global cognitive performance and executive function (Mungas et al., 2005; van den Heuvel et al., 2006; Kramer et al., 2007). In addition, WMH may also have a role as a surrogate marker to assess treatment efficacy. The impact of progression of WMH on stroke and dementia are also needed to help design therapeutic trials incorporating progression of WMH as an intermediate end point. In order to accurately observe the progression of WMH, the reproducibility of WMH measurement is of critical significance. The reliability of WMH quantification based on images acquiring from different scanners in multiple centers is of crucial importance in multi-center and follow-up studies. It is thought that a direct comparison of images or WMH quantities from different scanners in different centers may induce great variation, but no study examined the extent of this variation compared with within-center variability. The uncertain or lower reproducibility of WMH quantification across centers can contribute to a major concern for carrying out multicenter and longitudinal research, as well as clinical trials.

In this study, we carry out the study on the reproducibility of WMH quantification, which covers both intra-scanner and inter-scanner variability, 2D–3D magnetic resonance imaging (MRI) variability, MR system upgrade variability, and image processing tools variability in WMH quantification. The results of this study can provide great help and guidance in multicenter and longitudinal WMH study design.

## Materials and Methods

### Participants

Six post-stroke patients with last onset more than 6 months (4 male and 2 female; mean age = 62.8 years; range = 58–72 years) were prospectively recruited from the outpatient clinic at the Department of Neurology, the First Hospital of Jilin University, P.R. China. Exclusion criteria were cortical infarction > 1/3 hemisphere, severe neuropsychiatric disorders, and a history of traumatic brain injury or tumors. In addition, to exclude the confounding effect of edema, all the participants had been without treatment with dehydrating agent or steroid within 4 weeks before MRI scans. Based on visual assessment of WM lesions, Fazekas scale was assessed on all 3D T2-FLAIR images by an experienced radiologist (CJG), and the mean Fazekas scale score of each subject was recorded. The median Fazekas scale score of all participants was 2.2 (range 1–3) (Fazekas et al., 1987). The study was approved by the local ethics committee and written informed consent was obtained from all participants.

### Image Acquisition

All participants were scanned within 12 h across four clinical MRI systems: MR1: 1.5-T Siemens Avanto (software: syngo MR B15); MR2: 1.5-T Siemens Avanto (software: syngo MR B17) (Siemens Healthcare, Erlangen, Germany); MR3: 3.0-T Philips Ingenia (Philips Healthcare, Best, the Netherlands), and MR4: 3.0-T Siemens Trio (Siemens Healthcare, Erlangen, Germany). 3D T1-weighted MRI sequence was obtained for assisting accurate WMH segmentation. 3D T2-FLAIR and 2D T2-FLAIR were acquired twice with repositioning in-between on each MRI system, resulting in a total of 16 T2-FLAIR volumes per participant. All the 2D T2-FLAIR parameters were from the default clinical sequences. The MRI acquisition parameters are detailed in Table 1.

**TABLE 1:** MRI acquisition parameters.

	**MR1**	**MR2**	**MR3**	**MR4**
**Manufacturer**	Siemens	Siemens	Philips	Siemens
Model name	Avanto	Avanto	Ingenia	TrioTim
Station name	MEDPC26921	MRC25494	3FCD991	MRC35363
System version	syngo MR B15	syngo MR B17	R6.0.531.1	syngo MR B15
Field strength (T)	1.5	1.5	3	3
**2D FLAIR**				
Voxel size, mm^3^	0.5 × 0.5 × 6.0	0.5 × 0.5 × 6.0	0.5 × 0.5 × 6.0	0.5 × 0.5 × 6.0
Number of slices	20	20	18	20
Repetition time (ms)	9,000	9,000	7,000	8,000
Echo time (ms)	99	103	93	93
Flip angle (°)	150	150	90	130
Voxel size, mm^3^	1.0 × 1.0 × 1.0	1.0 × 1.0 × 1.0	1.0 × 1.0 × 1.0	1.0 × 1.0 × 1.0
Number of slices	176	176	344	176
Repetition time (ms)	7,500	7,500	4,800	7,500
Echo time (ms)	402	396	310	389
Flip angle (°)	120	120	90	120
**3D T1WI**				
Voxel size, mm^3^	1.0 × 1.0 × 1.0	1.0 × 1.0 × 1.0	1.0 × 1.0 × 1.0	1.0 × 1.0 × 1.0
Number of slices	176	176	192	176
Repetition time (ms)	1,900	1,900	7.07	1,900
Echo time (ms)	3.37	3.37	3.19	2.96
Flip angle (°)	15	15	7	9

*FLAIR, fluid-attenuated inversion recovery; T1WI, T1-weighted images.*

### Image Processing

Two fully automated WMH segmentation and quantification software were used for WMH segmentation and volumetric measurement. One is AccuBrain^®^ (BrainNow Medical Technology Ltd.) and the other is lesion growth algorithm [16] as implemented in the Lesion Segmentation Toolbox (LST^1^). AccuBrain^®^ is an automated brain segmentation and quantification software. It can segment a list of brain structures based on T1w MRI. Given additional T2-FLAIR MRI, it can also segment and quantify WMH (Shi et al., 2013). AccuBrain^®^ segments T1w MRI and produces brain structure masks and tissue masks. Then, it coregisters T1w MRI with T2-FLAIR MRI and transforms the structure and tissue masks onto T2-FLAIR space. Using a set of morphological techniques, it extracts WMH on T2-FLAIR MRI and refines it using the transformed brain structure mask from T1w MRI. AccuBrain^®^ is a cloud-based computing tool, which only requires MRI scans as input with no other tunable parameters.

LST is an open source toolbox of SPM used to segment T2 hyperintense lesions in FLAIR images. LST also relies on both T1w and T2-FLAIR MRI to segment WMH. It determines the three tissue classes of gray and white matter as well as cerebrospinal fluid from the T1w MRI and then uses the T2-FLAIR intensity distribution of each tissue class to detect outliers. The neighboring voxels are analyzed and assigned to lesions under certain conditions. This is done iteratively until no further voxels are assigned to lesions. Herein, the likelihood of belonging to WM or GM is weighed against the likelihood of belonging to lesions. We used the default parameters in LST toolbox, initial threshold: 0.3, MRF parameter: 1, and maximum iterations: 50.

### Reproducibility Analysis

To measure the reproducibility, several metrics, i.e., volume difference percentage, CV, DSC, and ICC, were computed. Volume difference percentage is defined as the percentage of quantified WMH volume difference between the two sequential scans of the average WMH volume value of the two scans:

$$\mathrm{volume}\,\mathrm{difference}\,\mathrm{percentage}=\frac{\left|s\,c\,a\,n-r\,e\,s\,c\,a\,n\right|}{\left(s\,c\,a\,n+r\,e\,s\,c\,a\,n\right)/2}\times 100\%$$

CV is defined as the ratio of the standard deviation to the mean of the multiple measurements and is expressed in percentages.

$$C\,V=\frac{\sigma }{\mu }\times 100\%$$

DSC is defined as the volume overlap of two segmentations:

$$D\,S\,C\,\left(A,B\right)=\frac{2\,\left(A\cap B\right)}{\left|A\right|+\left|B\right|}$$

In this study, we first aligned all the WMH results in the MR1 3D FLAIR MRI space, used the STAPLE algorithm (Warfield et al., 2004) to combine the WMH segmentation labels of all the scans, and created a fused label as reference label; each segmentation label was compared with the reference label in terms of DSC.

ICC was computed using two-way mixed method with 95% CIs in IBM SPSS Statistics 20 software.

The reproducibility of WMH quantification was assessed from four aspects.

#### Intra-Scanner Reproducibility

Each subject has a set of scanned 3D T1w, 2D T2-FLAIR, and 3D T2-FLAIR MRIs and re-scanned 2D T2-FLAIR and 3D T2-FLAIR MRIs on each MR scanner. The set of scan–rescan T2-FLAIR MRIs were used for examining within-scanner repeatability in a single center. The volume difference percentage, CV, DSC, and ICC between two sequential WMH measures using scan–rescan images were computed.

#### Inter-Scanner Reproducibility

The studying subjects were scanned with the same set of image sequences (3D T1w, 2D T2-FLAIR, and 3D T2-FLAIR) across four different scanners within 12-h interval. The inter-scanner variability was evaluated using CV, DSC, and ICC values of the same subject’s different WMH measurements.

#### MR System Software Variability

Two of the four studying MRI scanners (MR1 and MR2) are the same MRI system from the same vendor (Siemens Avanto) but different in MRI system software (syngo MR B15 and syngo MR B17) and settled place. The effects of MR system upgrade and examination place on WMH volume measurements were examined using this experiment.

#### 2D and 3D T2-FLAIR Variability

As each subject was scanned using both 2D and 3D T2-FLAIR on the same scanner, the WMH volume measurement difference between 2D and 3D T2-FLAIR images was studied.

## Results

### Segmentation

**FIGURE 1:**
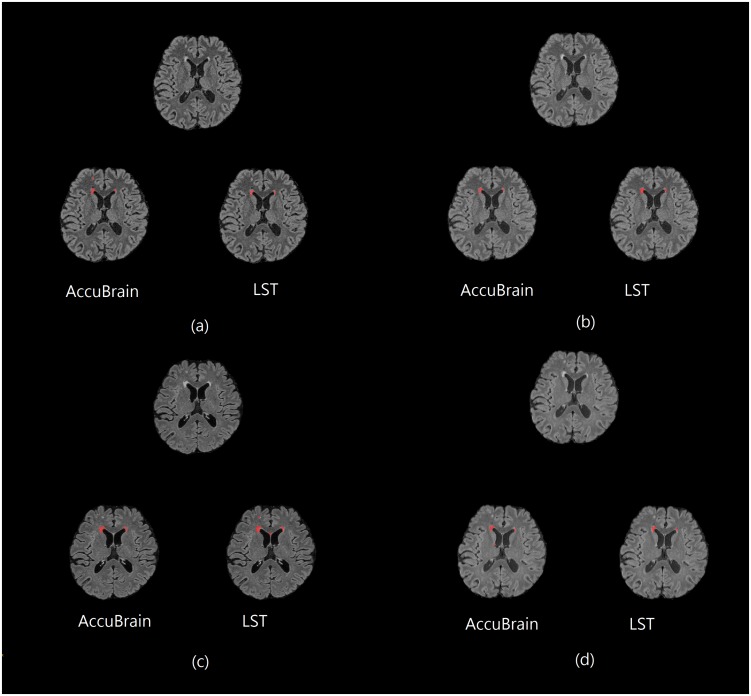
One subject’s 3D T2-FLAIR MR images from different scanners together with their WMH segmentation results (red overlay) using AccuBrain^®^ and LST. **(a)** MR1; **(b)** MR2; **(c)** MR3; and **(d)** MR4.

Figure 1 shows some representative axial slices of one subject’s 3D T2-FLAIR MR images from different acquisitions and their corresponding automatic WMH segmentation results of the two software. It can be observed that the T2-FLAIR MRIs have a large variability in appearance across scanners, which brings great challenge in obtaining consistent WMH volumetric measurement.

**FIGURE 2:**
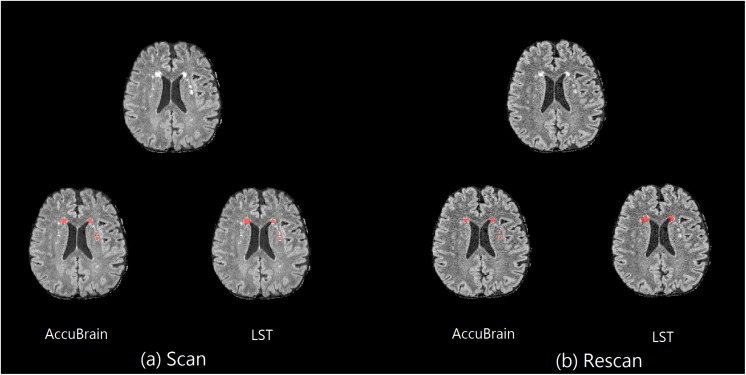
Scan–rescan example on MR1. The corresponding T2-FLAIR MRI slice from a 3D T2-FLAIR MRI scan–rescan experiment on MR1 scanner, together with their WMH segmentation results using AccuBrain^®^ and LST. **(a)** The first 3D T2-FLAIR scan. **(b)** Rescan with the subject’s position change.

In addition, on the same scanner, the subject’s imaging position change can also have an impact on T2-FLAIR MRI appearance and WMH segmentation results. One example can be seen in Figure 2, where images from a 3D T2-FLAIR scan–rescan test are shown. Even if the same scanner and imaging parameters are used within a short time period, the T2-FLAIR MRIs look different in tissue and WMH contrast.

**FIGURE 3:**
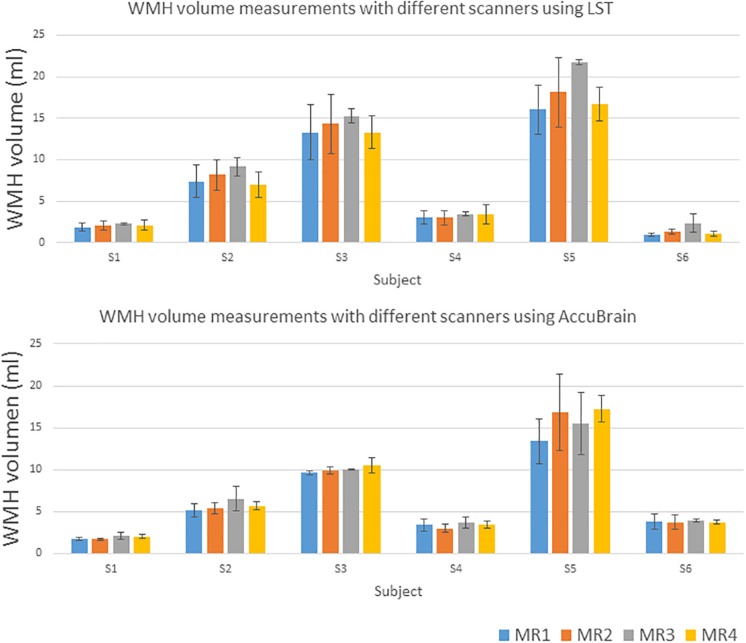
WMH volume measurements using LST and AccuBrain^®^ with MRIs from different scanners.

**FIGURE 4:**
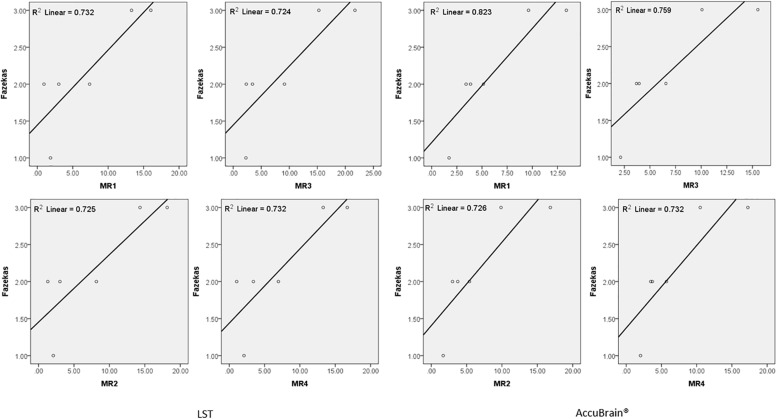
The correlation between Fazekas score and WMH volume measurements using LST and AccuBrain^®^ with MRIs from different scanners.

We quantified all the subjects’ segmented WMH using both LST and AccuBrain^®^ with all the MRIs from different scanners, as shown in Figure 3. The WMH volumes range from 1 to 20 ml for different subjects. We quantified all the subjects’ segmented WMH using both LST and AccuBrain^®^ with all the MRIs from different scanners, as shown in Figure 3. The WMH volumes range from 1 to 20 ml for different subjects. S1–S6’s mean Fazekas scale score is 1, 2, 3, 2, 3, and 2, respectively. The Pearson correlation between mean Fazekas scale score and LST quantified WMH volumes is 0.856 (MR1), 0.851 (MR2), 0.851 (MR3), and 0.856 (MR4), while the correlation is 0.907 (MR1), 0.852 (MR2), 0.871 (MR3), and 0.856 (MR4) with AccuBrain^®^ quantified WMH volume, as shown in Figure 4.

### Reproducibility

Table 2 shows the intra-scanner reproducibility results in the scan–rescan experiments. In general, the intra-scanner reproducibility results of different segmentation methods show relatively small differences with a mean volume difference percentage of 3.81% (3D) and 7.35% (2D) using AccuBrain^®^, and 4.20% (3D) and 8.07% (2D) using LST, and mean CV is 2.69% (3D) and 5.19% (2D) using AccuBrain^®^, and 2.97% (3D) and 5.71% (2D) using LST. The mean DSC is 0.73 (3D) and 0.68 (2D) using AccuBrain^®^, and 0.74 (3D) and 0.68 (2D) using LST. Comparatively, using 3D T2-FLAIR MRI brings more consistent WMH quantification results than using 2D-FLAIR MRI.

**TABLE 2:** Intra-scanner WMH volume measurement reproducibility using different image processing software.

		**Volume difference percentage (%) (mean ± std)**		**CV (%) (mean ± std)**		**DSC (mean ± std)**		**ICC (95% CI)**	
**3D**	**Scanner**	**AccuBrain^®^**	**LST**	**AccuBrain^®^**	**LST**	**AccuBrain^®^**	**LST**	**AccuBrain^®^**	**LST**
	All	3.81 ± 2.97	4.20 ± 5.15	2.69 ± 2.10	2.97 ± 3.64	0.73 ± 0.06	0.74 ± 0.07	0.996 (0.992–0.998)	1 (0.999–1)
	MR1	4.35 ± 0.15	7.25 ± 0.11	3.07 ± 1.98	5.12 ± 6.34	0.70 ± 0.04	0.73 ± 0.10	0.995 (0.965–0.999)	0.999 (0.994–1)
	MR2	3.36 ± 0.10	2.17 ± 0.11	2.37 ± 2.80	1.53 ± 0.74	0.74 ± 0.09	0.70 ± 0.06	0.999 (0.996–1)	1 (0.998–1)
	MR3	4.13 ± 0.21	2.40 ± 0.14	2.92 ± 2.58	1.70 ± 1.38	0.77 ± 0.04	0.75 ± 0.05	0.992 (0.943–0.999)	1 (0.998–1)
	MR4	3.41 ± 0.11	4.97 ± 0.07	2.41 ± 1.19	3.51 ± 2.82	0.73 ± 0.05	0.77 ± 0.05	0.999 (0.989–1)	0.999 (0.996–1)
***2D***	All	7.35 ± 5.86	8.07 ± 8.06	5.19 ± 4.14	5.71 ± 5.70	0.68 ± 0.10	0.68 ± 0.12	0.969 (0.930–0.986)	0.997 (0.993–0.999)
	MR1	4.11 ± 2.98	6.48 ± 4.77	2.90 ± 2.11	4.58 ± 3.37	0.67 ± 0.12	0.63 ± 0.14	0.998 (0.989–1)	0.999 (0.990–1)
	MR2	9.68 ± 7.35	10.56 ± 13.32	6.84 ± 5.20	7.46 ± 9.42	0.68 ± 0.12	0.70 ± 0.15	0.980 (0.866–0.997)	0.998 (0.982–1)
	MR3	10.1 ± 6.39	10.76 ± 7.67	7.14 ± 4.52	7.60 ± 5.42	0.65 ± 0.08	0.70 ± 0.09	0.959 (0.741–0.994)	0.995 (0.968–0.999)
	MR4	5.51 ± 4.65	4.48 ± 2.31	3.89 ± 3.29	3.16 ± 1.63	0.69 ± 0.08	0.67 ± 0.09	0.981 (0.871–0.997)	0.998 (0.985–1)

*FLAIR, fluid-attenuated inversion recovery; CV, coefficient of variation; DSC, Dice similarity coefficient; ICC, intra-class coefficient; 95% CI, 95% confidence interval.*

Table 3 compares the WMH volume measurement variability across different scanners. It shows that inter-scanner CV values are much larger than those of the intra-scanner experiment. Moreover, variability can also come from image processing software, where AccuBrain^®^ has an average inter-scanner CV value of 10.54% (3D), while LST’s inter-scanner CV value is 29.36% (3D) on average. In addition, MR1 and MR2 are two MRI scanners of the same type (Siemens Avanto) but with different versions of software installed and different rooms to be settled. There are still some variations between the quantifications on the two machines, but much smaller than that from different types of scanners.

**TABLE 3:** Inter-scanner WMH volume measurement reproducibility using different image processing software.

	**CV**				**DSC**	**ICC**
	**All scanners**	**95% CI of the difference**	**MR1 vs. MR2**	**95% CI of the difference**	**All scanners**	**All scanners**
**3D T2-FLAIR**						
AccuBrain^®^	10.54 ± 4.09	(6.239–14.84)	5.01 ± 2.35	(2.538–4.485)	0.64 ± 0.082	0.985 (0.947–0.998)
LST	29.36 ± 24.37	(3.785–54.95)	6.97 ± 4.29	(2.466–11.47)	0.62 ± 0.129	0.950 (0.837–0.992)
**2D T2-FLAIR**						
AccuBrain^®^	11.49 ± 4.62	(6.646–16.34)	8.37 ± 5.41	(2.683–14.05)	0.63 ± 0.079	0.967 (0.888–0.995)
LST	10.89 ± 4.81	(5.836–15.95)	11.39 ± 7.61	(3.401–19.38)	0.65 ± 0.118	0.985 (0.949–0.998)

*FLAIR, fluid-attenuated inversion recovery; CV, coefficient of variation; DSC, Dice similarity coefficient; ICC, Intra-class coefficient; 95% CI, 95% confidence interval.*

In Table 4, the differences between 2D T2-FLAIR and 3D T2-FLAIR MRI were calculated and compared. It can be observed that in both intra-scanner and inter-scanner settings, the WMH volume measurement variations between 2D and 3D MRI are large and vary across different image processing tools.

**TABLE 4:** Comparison of WMH quantification based on 2D and 3D T2-FLAIR.

	**Intra-scanner CV**		**Inter-scanner CV**	
	**Mean ± std**	**95% CI of the difference**	**Mean ± std**	**95% CI of the difference**
AccuBrain^®^	15.62 ± 8.73	(11.93–19.31)	17.17 ± 5.81	(11.07–23.27)
LST	24.19 ± 11.82	(19.19–29.18)	29.33 ± 15.01	(13.57–45.08)

*CV, coefficient of variation; 95% CI, 95% confidence interval.*

## Discussion

In this study, we examined the reproducibility of WMH quantification. To achieve this, subjects with different levels of WMH load have undertaken MRI acquisitions (3D T1w, 2D and 3D T2-FLAIR sequences) across four different MRI scanners. On each scanner, a scan–rescan procedure was performed to examine intra-scanner variability, while the inter-scanner variability was tested across the four scanners. Meanwhile, the effect of software upgrade and settled place was examined using the same type of scanner but installed with different versions of software and in different examination rooms. In addition, comparison of WMH volume measurements between 2D and 3D T2-FLAIR was also made in both intra-scanner and inter-scanner setting.

In the intra-scanner experiments, it has shown that 3D T2-FLAIR MRIs generally achieved much better reproducibility than 2D T2-FLAIR MRIs regardless of image processing software, where the between-scan volume difference percentage is 3.81–4.20% for 3D T2-FLAIR and 7.35–8.07% for 2D T2-FLAIR. The larger variability of 2D scans indicates that the large slice thickness of 2D MRI scan can bring large variation in WMH volume measurement due to the irregular pattern of WMH across slices. An average 4% volume difference percentage was achieved in the scan–rescan procedure using 3D T2-FLAIR MRI. This implies that for a subject with low WMH load, e.g., 2 ml, a deviation of 0.08 ml may be induced on average with the same imaging setting; meanwhile, for a subject with high WMH load, e.g., 10 ml, an average of 0.4 ml deviation may be induced. The scan–rescan reproducibility results can provide important clinical information in aiding doctor’s further assessment or diagnosis.

There are several existing works studying the within-center reproducibility WMH volumetric measurement using a scan–rescan procedure in a single center. For example, de Boer et al. (2010) assessed whiter matter lesion segmentation reproducibility by comparing the automatic segmentations (by trained kNN method) of 30 subjects who were scanned twice within a short time interval; the mean CV is 5.87% using 3D T1w and 3D T2-FLAIR MRI. Another study assessed reproducibility of three automated segmentation pipelines for quantitative MRI measurement of brain white matter signal abnormalities (WMSA) on 30 subjects who were positioned and imaged twice within 30 min and achieved a range of 2.57–7.76% CV values using different pipelines (Wei et al., 2002). Ramirez et al. evaluated Lesion Explorer (LE), an MRI-derived tissue segmentation and brain region parcellation processing pipeline for obtaining intracranial tissue and subcortical hyperintensity volumetry in a short-term scan–rescan reliability test on 20 volunteers, with a reported intra-class correlation coefficient (ICC) of 0.9998 for subcortical hyperintensity measurement (Ramirez et al., 2013). In general, our reported intra-scanner CV values [CV: 2.69%, ICC: 0.996 (0.992–0.998) using AccuBrain^®^ and CV: 2.97%, ICC: 1 (0.999–1) using LST] are close to the reported indices in a previous study. However, the previous studies mainly focused on 3D T2-FLAIR MRI. As the commonly used protocol in clinical practice, WMH quantification based on 2D T2-FLAIR MRI is also of great interest to clinicians. It is validated in our experiments that CV values of WMH quantification based on 2D MRI is in the range of 2.90–7.60% using different MRI scanners and processing software. In the inter-scanner experiments, the inter-scanner CV values (10.54% using AccuBrain^®^ and 29.36% using LST) are around four to six times of the intra-scanner CV values (2.69% using AccuBrain^®^ and 2.97% using LST). The large inter-scanner variability is mainly due to various T2-FLAIR MRI appearances resulting from different imaging parameters on different scanners. If the same scanner and imaging parameters are used, the difference can be smaller, where 5.01% and 6.97% CV value was achieved with AccuBrain^®^ and LST, respectively, using the same Siemens Avanto scanner but with different versions of software and installation places. This variability is in the same level of intra-scanner variability, implying that machine software upgrade and installation place can have little impact on the measurement of WMH volume. However, it still suggests that centers should consider having some assessment or calibration for quality assurance and to calculate differences across time when scanner upgrade or replacement are considered.

In comparing quantification using both 2D and 3D T2-FLAIR, it has revealed that the variability is quite high under both intra-scanner and inter-scanner setting. 2D T2-FLAIR MRI is commonly accepted in clinical practice for diagnosis or assessment due to its relatively short acquisition time. However, WMH quantification results based on 2D MRI cannot be directly compared with the 3D MRI quantities, even with some resampling techniques, as it is easy to underestimate or overestimate WMH volume using 2D MRI.

Recommendations for multicenter WMH quantitative study:

(1) Acquire 3D T2-FLAIR MRIs using the same imaging parameters on the same scanner. Intra-scanner 3D T2-FLAIR reproducibility is much higher than others regardless of automatic quantification tools. Followed is the reproducibility of the same scanner but with upgraded software and resettled place. It indicates that in order to achieve the highest reproducibility, acquiring 3D T2-FLAIR MRIs in the same scanner is preferred in a multicenter study or a longitudinal study.
(2) WMH quantification on 2D T2-FLAIR MRIs is not comparable with that on 3D T2-FLAIR MRIs. Due to large slice thickness and irregular WMH pattern across slices, the variability of WMH volumetric measurement based on 2D T2-FLAIR MRI is much larger than 3D T2-FLAIR MRI. Although 2D T2-FLAIR MRI is commonly used in clinical practice, it is not preferable in a multicenter study or follow-up comparison. In particular, a direct comparison of quantitative results between 2D and 3D MRI can result in large deviation.
(3) WMH segmentation methods have a large impact on the quantification results and reproducibility. It can be observed that the scan–rescan reproducibility is relatively stable among different segmentation tools. However, the inter-scanner reproducibility is various among different tools. Choosing and comparing different image processing software is also an important issue in reliable WMH measurement.
(4) Before multicenter clinical trial is carried out, if different scanners are involved, protocol optimization and harmonization should be implemented first in each scanner. A reproducibility experiment with phantom or volunteer assessments for quality assurance is important to calculate differences in brain quantification.
(5) In a multicenter study, when images from different scanners have already been acquired, it is advised to (1) choose proper WMH quantification tools that are designed robust to scanner change and (2) use dedicated statistical models to adjust on scanner or use random-effects models.

Our study has several limitations. First, the subject number is not yet large enough for us to draw a statistically meaningful conclusion. Due to the long acquisition time for each subject to complete the whole procedure within 1 day, it is difficult to recruit many subjects in the current study. Second, MRI data acquisition was held in a single center. Although inter-scanner acquisitions were performed on different scanners and in different rooms to simulate multi-center study design, it is necessary in the future to launch multi-center reproducibility study based on a large population.

## Conclusion

In conclusion, we compared WMH quantification reproducibility in different experimental settings. In general, the reproducibility was the best when performing WMH segmentation on 3D MRI acquired by the same type of MRI scanner and same imaging parameters regardless of automatic segmentation tools. This study gives evidence on the extent of variability of WMH measurement across centers and can also aid in designing multicenter and longitudinal study to have enough power.

## Ethics Statement

This study was carried out in accordance with the recommendations of the First Hospital of Jilin University, China local ethics committee with written informed consent from all subjects. All subjects gave written informed consent in accordance with the Declaration of Helsinki. The protocol was approved by the First Hospital of Jilin University, China ethics committee.

## Author Contributions

CG, KN, YL, LSh, HZ, and LSu contributed to the study concept and design. CG, ZW, and MZ contributed to acquisition of the data. CG, KN, YL, LSh, DW, WZ, HZ, and LSu contributed to analysis and interpretation. CG, KN, YL, LSh, HZ, and LSu drafted the manuscript. All authors contributed to the critical revision of the manuscript for important intellectual content.

## Conflict of Interest Statement

LSh was the director of BrainNow Medical Technology Limited. YL was an employee of BrainNow Medical Technology Limited, which developed AccuBrain^®^ used in this manuscript. The remaining authors declare that the research was conducted in the absence of any commercial or financial relationships that could be construed as a potential conflict of interest.

## Footnotes

1 https://www.applied-statistics.de/lst.html

## Abbreviations

95% CI: 95% confidence interval
CV: coefficient of variation
DSC: Dice similarity coefficient
FLAIR: fluid-attenuated inversion recovery
ICC: intra-class coefficient
SPM: statistical parametric mapping
WMH: white matter hyperintensities.
